# Comparison of manual and automated respiratory rate measurements on hospital wards: a prospective observational study

**DOI:** 10.1007/s10877-025-01380-1

**Published:** 2025-11-15

**Authors:** Sherif Gonem, Lucy Stones, Donna Ward, Steve Briggs, Tricia McKeever

**Affiliations:** 1 Department of Respiratory Medicine, Nottingham University Hospitals NHS Trust, Hucknall Road, NG5 1PB Nottingham, UK; 2 NIHR Nottingham Biomedical Research Centre, School of Medicine, University of Nottingham, Nottingham, UK; 3 PMD Solutions, Cork, Republic of Ireland; 4 Digital and Information, Nottingham University Hospitals NHS Trust, Nottingham, UK

**Keywords:** Wearable device, Continuous monitoring, Respiratory rate, Early warning score

## Abstract

Respiratory rate is an important early sign of clinical deterioration but the current practice of counting breaths manually is time-consuming and prone to error. We aimed to determine the concordance between manual respiratory rate measurements and automated measurements recorded using a wearable device. We undertook a prospective observational study on three general respiratory wards to compare manual respiratory rate measurements collected during usual clinical care with automated readings from a wearable respiratory rate monitor (RespiraSense, PMD Solutions, Cork, Ireland). Thirty-one patients took part in the study. Manual respiratory rate readings displayed large peaks at 20 and 24 breaths/min, whereas automated readings followed a smooth bell-shaped distribution. Manual and automated respiratory rates were both higher during the day than at night, and this was more marked for automated readings. Automated readings were on average 2.5 (95% confidence interval [CI] 2.2 to 2.8) breaths/minute higher than time-matched manual readings, and the 95% limits of agreement were − 7.9 (95% CI -8.4 to -7.4) and 12.9 (95% CI 12.3 to 13.4) breaths/minute, wider than the clinically acceptable limits of ± 3 breaths/min. Trends in manual and automated respiratory rates were concordant in only 56% of cases. Automated respiratory rate measurements using RespiraSense do not display clinically acceptable agreement with manual measurements in the setting of a respiratory ward.

**Supplementary Information:**

The online version contains supplementary material available at 10.1007/s10877-025-01380-1.

## Introduction

Respiratory rate is an important early marker of clinical deterioration and can be an indicator impending cardiac arrest [1]. It is also an important component of the National Early Warning Score-2 (NEWS-2), the scoring system which is used throughout the UK to detect deteriorating patients in hospital [2]. However, previous studies have shown that manual assessment of respiratory rate using visual observation is often inaccurate [3]. Moreover, intermittent measurement of respiratory rate every 4–6 h could miss the opportunity to detect patient deterioration that occurs in the intervening period. A number of technologies have been developed for measuring respiratory rate objectively, including both wearable and contactless devices [4].

RespiraSense (PMD Solutions, Cork, Republic of Ireland) is a commercial medical device for continuously monitoring respiratory rate [5, 6]. It consists of a single-use adhesive patch connected to a reusable plastic lobe, and is worn by patients at the boundary between their lower ribs and upper abdomen. The adhesive patch incorporates two piezoelectric deformation transducers that detect periodic chest wall and abdominal movement associated with breathing. This is connected to a reusable plastic lobe that incorporates a processor, an accelerometer and a Bluetooth transmitter. The signal from the accelerometer is used to detect motion artefacts, and this is combined with signals from the piezoelectric transducers to produce a motion-corrected respiratory waveform, from which the respiratory rate is calculated. The real-time respiratory rate readings are transmitted to a central monitoring station by the Bluetooth transmitter.

Respiratory rate measured using RespiraSense showed good concordance with manual respiratory rate measurements in a post-anaesthesia care unit [7] and an acute medical unit [8]. However, these studies were carried out under controlled conditions with relatively short periods of observation. There have been no previous studies evaluating RespiraSense in a general ward setting with prolonged monitoring.

We undertook a prospective observational study to compare manual and automated respiratory rate measurements in a general ward setting. The primary aim of the study was to determine the degree of concordance between manual and automated measurements. We also aimed to describe the distributions of manual and automated respiratory rates and their diurnal variability.

## Methods

### Setting

The study was carried out on three medical respiratory wards at Nottingham City Hospital, a large acute hospital in the East Midlands region of the UK.

### Population

Patients were selected for RespiraSense monitoring by ward nursing staff if they were considered to be at risk of respiratory deterioration. This included patients requiring supplementary oxygen, those with a National Early Warning Score-2 (NEWS-2) of ≥ 5, and those with a condition that had the potential to deteriorate such as pneumonia, Covid-19 or acute exacerbation of chronic obstructive pulmonary disease. Patients who were selected for monitoring by the clinical team were then approached to take part in this observational study. Participants gave their written informed consent for their clinical and RespiraSense monitoring data to be extracted and analysed by the research team. Patients who chose not to take part in the study could still receive monitoring with RespiraSense for their own benefit, since this was a CE-marked commercially available device which was being used for its intended purpose.

### RespiraSense monitoring

The RespiraSense patch was applied to the lower chest/upper abdomen according to the manufacturer’s instructions. Continuous respiratory rate data were transmitted to a central monitoring console and could be viewed in real-time by ward nursing staff. Alarms were initially set to sound if the respiratory rate exceeded 24 breaths/minute or fell below 10 breaths/minute. Alarm settings could be manually altered by staff, who were advised to set the upper alarm limit at 4 breaths/minute above the previously recorded manual respiratory rate. Nurses were asked to respond to a RespiraSense alarm by immediately checking on the patient and carrying out a full set of clinical observations.

### Manual respiratory rate measurements

Manual respiratory rates were measured by ward nurses according to their usual practice as part of regular clinical observations. These were carried out at a frequency which ranged from every 12 h to every 30 min, depending on the patient’s level of acuity. Clinical observations were recorded electronically on the Nervecentre system as part of usual clinical care.

### Data collection

Date and time-stamped manual and automated respiratory rate measurements were extracted in a de-identified format for analysis, covering the period from 30th November 2022 to 11th March 2024. Manual and automated respiratory rates were extracted from the clinical Nervecentre system and the cloud-based RespiraSense database respectively.

### Statistical analysis

Statistical analysis was undertaken using IBM SPSS V28.0 and Microsoft Excel 2016, with the threshold for statistical significance set at *p* < 0.05. A Bland-Altman plot [9] was used to assess the concordance between paired manual and automated respiratory rate measurements that were taken within 15 min of each other. We defined clinically acceptable agreement as a mean difference and 95% limits of agreement both within ± 3 breaths/min, in line with previous literature [4]. A four-quadrant plot was used to assess concordance of trends over time, using the changes (Δ) between consecutive time-matched manual and automated respiratory rates in the same subject [10]. Concordance was defined as paired Δ manual respiratory rate and Δ automated respiratory rate both being ≥ 0 or both < 0. Paired Δs that were both between − 2 and 2 breaths per minute (inclusive) were excluded from the four-quadrant plot and concordance analysis, since these were considered to be clinically insignificant and within the measurement error of the readings.

### Sensitivity analysis

We repeated the Bland-Altman and four-quadrant analyses using a single randomly selected data point from each patient, in order to investigate the influence of multiple repeated within-patient measurements on our results. The random selection was carried out using the Excel RAND function.

### Sample size calculation

Bland-Altman analysis requires 200 paired samples to achieve excellent precision for the upper and lower limits of agreement [11]. Specifically, the 95% confidence intervals (CI) for the limits of agreement are +/- 0.24s for 200 paired samples, where s is the standard deviation of the differences between measurements by the two methods. Assuming that we would obtain 8 paired measurements per patient, we calculated that data from 25 patients would be sufficient to achieve excellent precision.

## Results

Thirty-one patients (45% female) gave written informed consent to take part in the study. The mean (standard deviation [SD]) age at admission was 72.4 (10.5) years. The median (interquartile range [IQR]) length of monitoring was 4.4 (2.2–8.5) days. 1121 paired manual and automated respiratory rate readings were obtained in total. These were closely matched in time, with the mean (SD) of the time difference being 3.5 (3.4) minutes. Detailed clinical and demographic characteristics of the study cohort are shown in Table 1.

**Table 1:** Clinical and demographic characteristics of study cohort

Clinical or demographic characteristic	Study cohort values (*n* = 31)
Age (years, mean [SD])	72.4 (10.5)
Sex (female : male [% female])	14:17 (45)
In-hospital mortality (died : survived [% died])	7:24 (23)
ICU admission (admitted : not admitted [% admitted])	2:29 (6)
*Primary diagnosis (n, %)* COPD exacerbation Pneumonia or LRTI Covid-19 Heart failure Pleural effusion Empyema Interstitial lung disease Obesity hypoventilation syndrome Neuromuscular disease Aspergillosis	7 (23) 6 (19) 5 (16) 3 (10) 3 (10) 2 (6) 2 (6) 1 (3) 1 (3) 1 (3)
Length of monitoring with RespiraSense (days, median [IQR])	4.4 (2.2–8.5)
Number of paired manual and automated respiratory rates (median [IQR])	31 (13–50.5)

*SD S*tandard deviation; *ICU I*ntensive care unit; *COPD C*hronic obstructive pulmonary disease; *LRTI *Lower respiratory tract infection; *IQR *Interquartile range

**Fig. 1:**
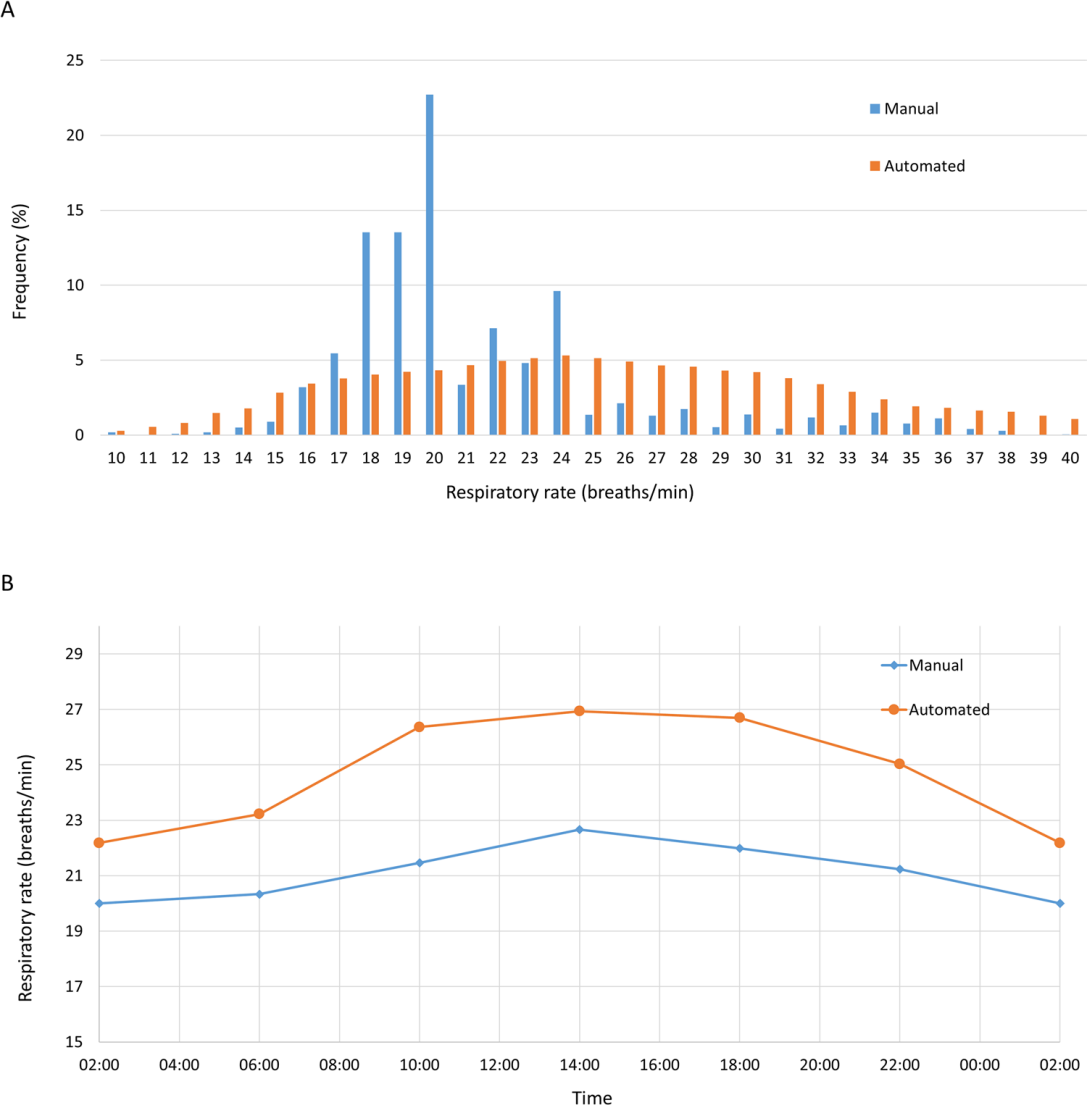
Frequency distribution and diurnal variability of manual and automated respiratory rate measurements. Panel A shows the frequency distribution of manual and automated respiratory rate measurements. Panel B shows the diurnal variability of the measurements

**Fig. 2:**
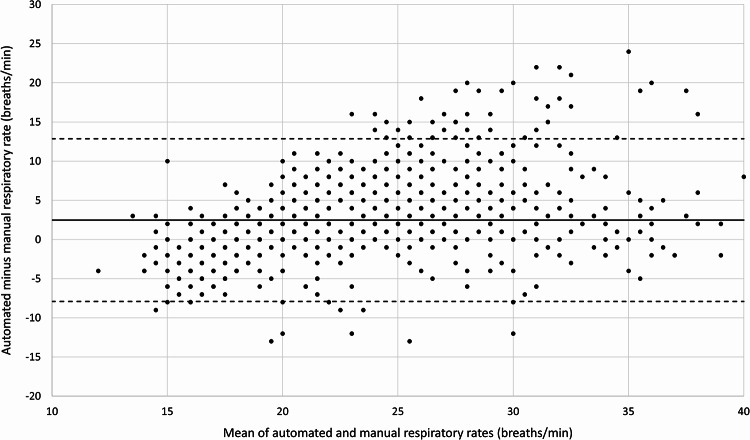
Bland-Altman plot showing concordance between paired manual and automated respiratory rate measurements. Mean bias and 95% limits of agreement are indicated by the solid and dotted lines respectively

**Fig. 3:**
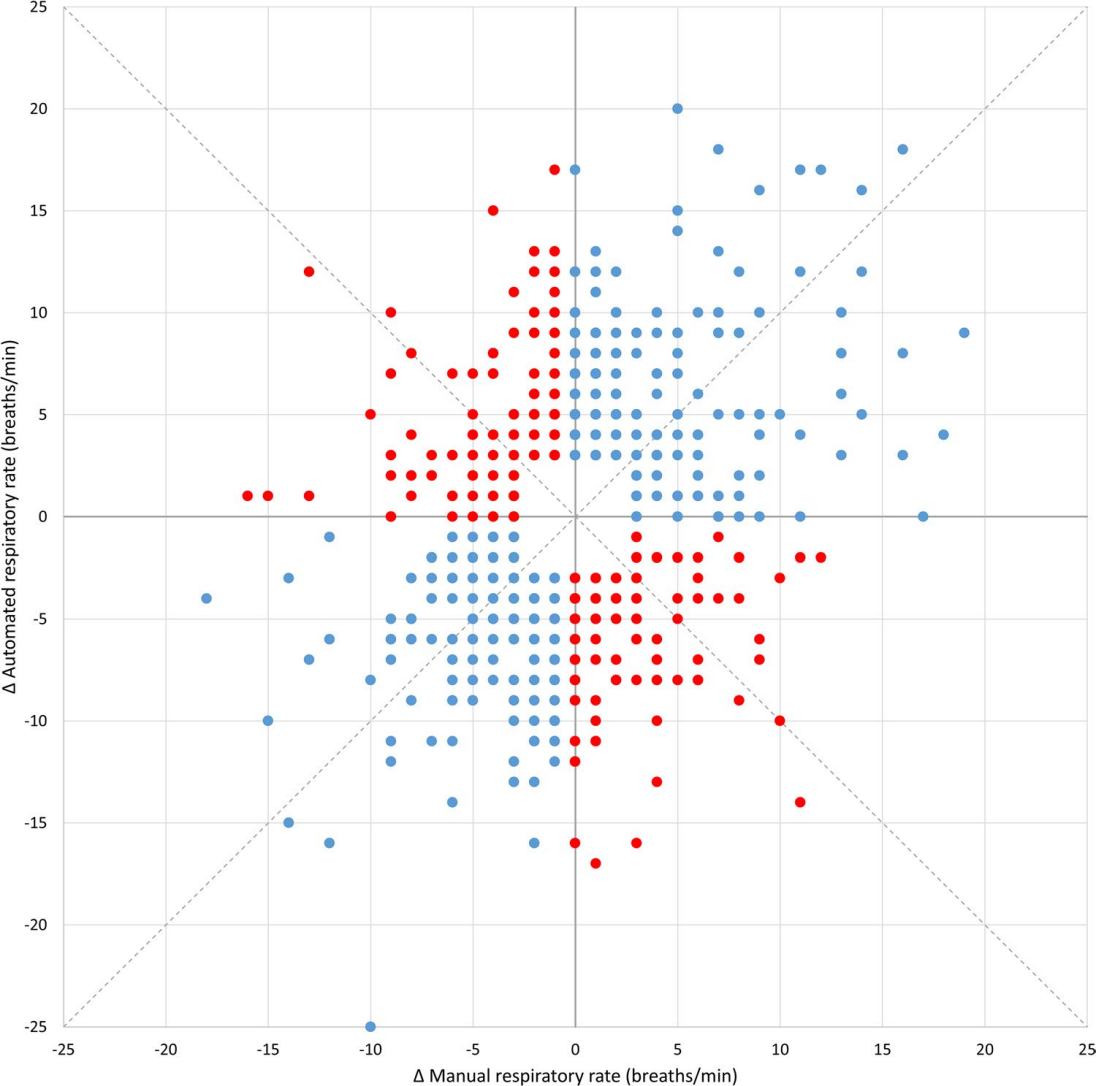
Four-quadrant plot showing concordance between changes (Δ) in paired manual and automated respiratory rate measurements

Figure 1a shows the distribution of manual and automated respiratory rate measurements. 75% of manual respiratory rate readings were in the range from 18 to 24 breaths/min, and 23% of manual readings were 20 breaths/min. Automated readings followed a smooth bell-shaped distribution, with a maximum at 24 breaths/minute. Both manual and automated respiratory rate readings displayed diurnal variability, with higher values during the day than at night (Fig. 1b). This was more marked for automated than for manual readings. Figure 2 shows a Bland-Altman plot of manual versus automated respiratory rate readings. Automated readings were on average 2.5 (95% CI 2.2 to 2.8) breaths/minute higher than time-matched manual readings, and the 95% limits of agreement were − 7.9 (95% CI −8.4 to −7.4) and 12.9 (95% CI 12.3 to 13.4) breaths/minute. Using a single data point from each participant, automated readings were on average 3.6 (95% CI 1.7 to 5.6) breaths/minute higher than time-matched manual readings (Supplementary Figure S1). The 95% limits of agreement were − 7.0 (95% CI −10.5 to −3.6) and 14.3 (95% CI 10.9 to 17.8) breaths/minute. Figure 3 shows a four-quadrant plot with Δ manual respiratory rate plotted against Δ automated respiratory rate. Concordance was only 56%, suggesting little or no relationship between trends measured using the two methods. Using a single data point from each participant, concordance was similarly low at 58% (Supplementary Figure S2).

### Discussion

We undertook a prospective observational study to compare manual respiratory rate readings taken in the course of usual clinical care with automated readings recorded by a commercially available wearable monitoring device.

Automated respiratory rate readings followed a smooth bell-shaped distribution, as might be expected of a physiological measurement. However, as has been reported before [12, 13], manual respiratory rate measurements displayed prominent peaks at specific values, namely 20 and 24 breaths/minute. In particular, 22.7% of manual respiratory rates were 20 breaths/minute whereas only 3.4% were 21 breaths/minute. This difference cannot be explained physiologically, and suggests that a significant proportion of manual respiratory rates may have been estimated rather than formally counted. It is also possible that the peaks at 20 and 24 were due to nurses counting breaths for 15 s and multiplying by 4. Furthermore, it is notable that crossing the boundary between a respiratory rate of 20 and 21 breaths/minute results in a two-point increase in the NEWS-2 score, which could lead to an obligation to call for medical assistance.

We observed that automated respiratory rate readings were on average 2.5 breaths/minute higher than time-matched manual measurements. This may have been due to a systematic over-reading of respiratory rate by RespiraSense or under-reading by ward nurses. Alternatively, the discrepancy could have arisen because manual measurements are generally made at rest, whereas automated readings may have captured periods of activity occurring just before or after the manual measurement. We also found that the 95% limits of agreement were wider than in previous studies using RespiraSense [7, 8]. This is likely to be due to the fact that our study was conducted in a real-life clinical setting rather than under controlled research conditions, potentially reducing the accuracy of both the automated and manual readings. The automated readings could have been affected by movement artefacts or sub-optimal placement of the wearable patch, while manual respiratory rate measurements carried out in the course of usual clinical care are known to have multiple sources of inaccuracy [3].

Our results are concordant with several previous studies that have compared manual and automated respiratory rates using a variety of devices [14–17]. In all cases, there was a low correlation between respiratory rates measured during usual clinical care and automated measurements. There has been conflicting evidence regarding whether manual or automated readings provide better prognostic information. Kellett et al. found that in acutely ill medical patients, automated readings were independent predictors of in-hospital mortality whereas manual respiratory rates were not [14]. In contrast, Churpek et al. found that manual respiratory rate readings were actually better predictors of Intensive Care Unit admission than automated readings. The investigators suggested that this could be because manual readings were adjusted up or down to incorporate additional information about nursing concern [15]. Nurses will often use additional clues to determine if a patient has respiratory compromise, including the depth and effort of breathing, and this information may have prognostic significance.

### Limitations

This study was primarily designed to assess the concordance between manual and automated respiratory rate measurements. The relatively small sample size and observational study design do not allow any conclusions to be drawn about whether automated respiratory rate monitoring leads to earlier detection of clinical deterioration or improved patient outcomes.

## Conclusions

Automated respiratory rate measurements using RespiraSense do not display clinically acceptable agreement with manual measurements in the setting of a respiratory ward. Further development of the RespiraSense system is likely to be needed before it will be suitable for widespread clinical use.

## Supplementary Information

Below is the link to the electronic supplementary material.

Supplementary Material 1

## Data Availability

Current ethical and institutional approvals do not permit data sharing. Requests to share data should be made to the corresponding author.
