# Supplementary material for: Comparison of manual and automated respiratory rate measurements on hospital wards: a prospective observational study
Source: J Clin Monit Comput. 2025 Nov 15;40(2):449–55. doi: 10.1007/s10877-025-01380-1 (PMC13053469; doi:10.1007/s10877-025-01380-1)

Comparison of manual and automated respiratory rate measurements on hospital wards: a prospective observational study

[1,2]Sherif Gonem PhD

[1,3]Lucy Stones BSc

[1]Donna Ward BSc

[4]Steve Briggs BSc

[2]Tricia McKeever PhD

[1]Department of Respiratory Medicine, Nottingham University Hospitals NHS Trust, Nottingham, UK.

[2]NIHR Nottingham Biomedical Research Centre, School of Medicine, University of Nottingham, Nottingham, UK.

[3]PMD Solutions, Cork, Republic of Ireland

[4]Digital and Information, Nottingham University Hospitals NHS Trust, Nottingham, UK

Supplementary results

Figure S1: Bland-Altman plot showing concordance between paired manual and automated respiratory rate measurements – sensitivity analysis using one data point per patient

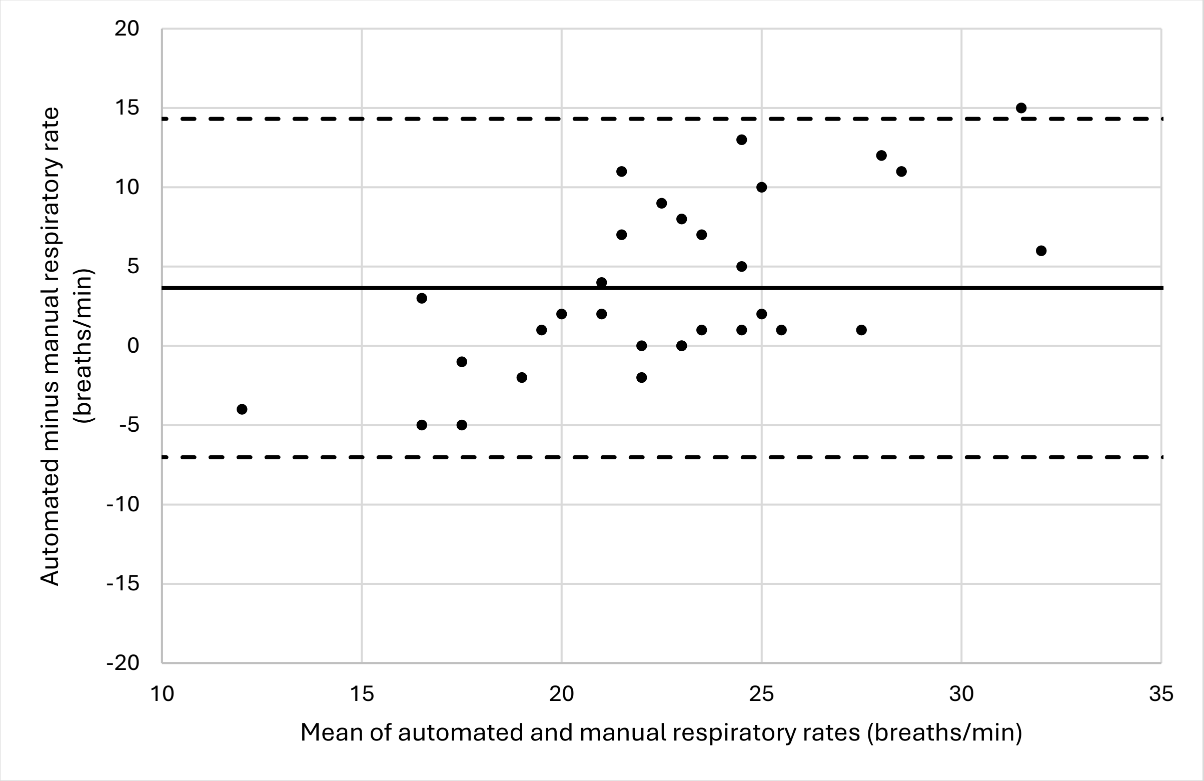

Mean bias and 95% limits of agreement are indicated by the solid and dotted lines respectively.

Figure S2: Four-quadrant plot showing concordance between changes (Δ) in paired manual and automated respiratory rate measurements – sensitivity analysis using one data point per patient

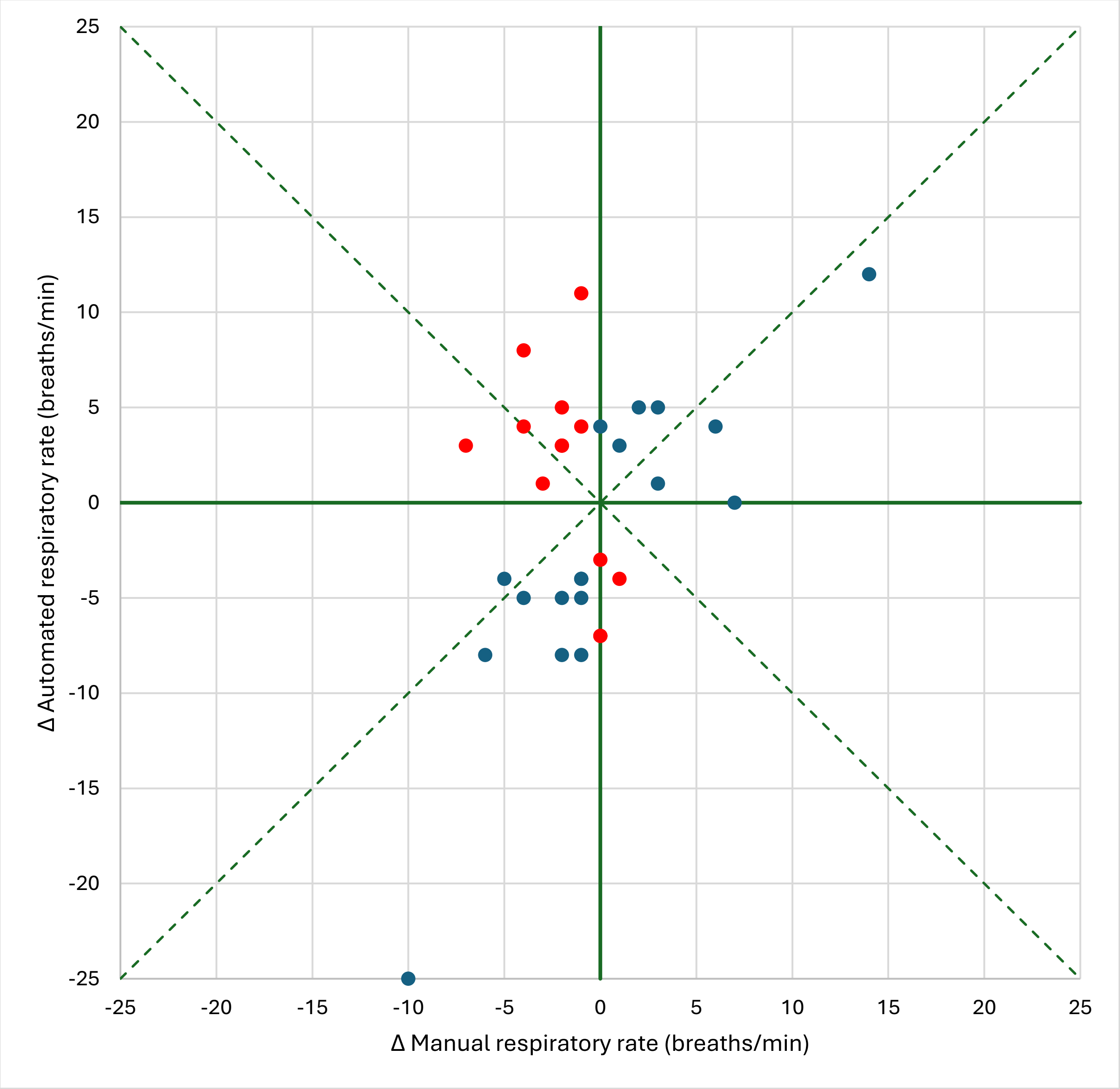

Supplement: Supplementary file 1 — Supplementary Material 1 [file 10877_2025_1380_MOESM1_ESM.pdf]
